# A New Method Based on CEEMD Combined With Iterative Feature Reduction for Aided Diagnosis of Epileptic EEG

**DOI:** 10.3389/fbioe.2020.00669

**Published:** 2020-06-30

**Authors:** Mengran Zhou, Kai Bian, Feng Hu, Wenhao Lai

**Affiliations:** ^1^School of Electrical and Information Engineering, Anhui University of Science and Technology, Huainan, China; ^2^State Key Laboratory of Mining Response and Disaster Prevention and Control in Deep Coal Mines, Anhui University of Science and Technology, Huainan, China

**Keywords:** intelligent diagnosis, EEG signals, complementary ensemble empirical mode decomposition, feature reduction, gray wolf optimizer

## Abstract

In the clinical diagnosis of epileptic diseases, the intelligent diagnosis of epileptic electroencephalogram (EEG) signals has become a research focus in the field of brain diseases. In order to solve the problem of time-consuming and easily influenced by human subjective factors, artificial intelligence pattern recognition algorithm has been applied to EEG signals recognition. However, at present, the common empirical mode decomposition (EMD) signal decomposition algorithm does not consider the problem of mode aliasing. The EEG features obtained by feature extraction may be mixed with some unimportant features that affect the classification accuracy. In this paper, we proposed a new method based on complementary ensemble empirical mode decomposition (CEEMD) combined with iterative feature reduction for aided diagnosis of epileptic EEG. First of all, the evaluation indexes of decomposing and reconstructing signals by several methods were compared. The CEEMD was selected as the decomposition method of the signals. Then, the support vector machine recursive elimination (SVM-RFE) was used to reduce 9 features extracted from EEG data. The support vector classification of the gray wolf optimizer (GWO-SVC) recognition model was established for different feature subsets. By comparing the classification accuracy of training set and test set of different feature subsets, and considering the complexity of the model reflected by the number of features selected by SVM-RFE, the analysis showed that the 6 feature subsets with fewer features and higher classification accuracy could reflect the key information of epileptic EEG. The accuracy of the training set classification was 99.38% and the test set was as high as 100%. The recognition time was only 1.6551 s. Finally, in order to verify the reliability of the algorithm proposed in this paper, the proposed algorithm compared with the classification model established by the raw EEG signals and the optimization model established by other intelligent optimization algorithms. It is found that the algorithm used in this paper has higher classification accuracy and faster recognition time than other processing methods. The experimental results show that CEEMD combined with SVM-RFE is feasible for rapid and accurate recognition of EEG signals, which provides a theoretical basis for the aided diagnosis of epilepsy.

## Introduction

Epilepsy is a chronic disease of nervous system disorder caused by abnormal discharge of brain neurons (Sheng et al., 2018). Worldwide, the number of epileptics has exceeded 50 million (Yang et al., 2011). The symptoms of epilepsy patients are usually sudden loss of consciousness, muscle convulsions, etc., which make epilepsy patients have a high mortality rate (Kobow et al., 2012), so their daily life has been greatly troubled. If the epilepsy of seizure type can be accurately identified and classified so that doctors can take reasonable treatment plans, it can help epileptics avoid the risk of disease in advance (Chen E. et al., 2018). Therefore, it is of great significance to strengthen the early diagnosis and late treatment of epilepsy.

The analysis of electroencephalogram (EEG) signals has the characteristics of high efficiency, small damage, and low cost. It has become the main clinical diagnosis method of epilepsy. This method needs experienced doctors to observe the high amplitude synchronous rhythms such as sharp wave and spike-wave in EEG during the epileptic seizure for a long time with the naked eye (Lévesque et al., 2017), which will not only consume a lot of energy but also may get wrong diagnosis results due to various uncertain factors. Therefore, it is necessary to develop a method of automatic recognition of epileptic EEG. In recent years, machine learning and deep learning algorithms have been widely applied in the biomedical and health field (Wang et al., 2019, 2020; Deng et al., 2020; Hu et al., 2020). Artificial intelligence combined with EEG has achieved good results in the diagnosis and prediction of epilepsy and other diseases. For example, Bajaj and Pachori (2013) used empirical mode decomposition (EMD) to decompose EEG signals and improved the classification accuracy of epilepsy detection by analyzing the first three natural mode function components. Puspita et al. (2017) extracted the mean, standard deviation and median statistical features of EEG data, and then used the back-propagation neural network (BNN) to establish the classification and recognition model of EEG data of epilepsy patients and achieved the best classification results. Cao et al. (2017) combined the short-time Fourier transform (STFT) with a convolutional neural network (CNN) and used the deep learning algorithm to avoid the process of manual feature selection in EEG recognition. The analysis steps of EEG mainly include preprocessing of raw signals, feature extraction, recognition, and classification. EMD is often used as the decomposition method of EEG signals. However, only one or some IMF components selected by subjective experience are taken as the research object, which cannot completely contain the useful information of the original signals, so the accuracy of EEG obtained is low, and it cannot effectively identify different types of EEG. Several typical EEG feature indexes are extracted directly for classification and recognition. This method cannot judge whether the extracted EEG features are all effective EEG feature indexes, which not only increases the recognition time but also affects the accuracy of classification.

Complementary ensemble empirical mode decomposition (CEEMD) is a signal decomposition method developed on the basis of empirical mode decomposition (EMD) (Muñoz-Gutiérrez et al., 2018), it has obvious advantages in dealing with non-linear and non-stationary signals. Satija et al. (2017) used a modified CEEMD algorithm to achieve automatic detection and classification of ECG noise. Chen and Hsiao (2018) used the CEEMD method to extract hidden signals from the respiratory inductance plethysmography (RIP) signals based on the frequency bands of different respiratory muscles. Amezquita-Sanchez et al. (2016) combined CEEMD with magnetoencephalography (MEG) to distinguish patients with mild cognitive impairment (MCI). Support vector machine recursive feature elimination (SVM-RFE) is a feature selection method, it can eliminate the feature information of low importance, and effectively remove the interference of redundant information (Tapia et al., 2012), which is conducive to the establishment of the classification model. SVM-RFE has been widely used in biomedical research. Ding et al. (2015) proposed a method of SVM-RFE combined with voxel-based morphometry (VBM) to analyze MRI data and realized the automatic classification of smokers and non-smokers. Anaissi et al. (2016) used the ensemble SVM-RFE algorithm to select the characteristic genes in the genomic data. Bisdas et al. (2018) adopted the SVM-RFE method to select the most discriminative diagnostic biomarkers. Gray wolf optimizer (GWO) is a new swarm intelligent optimization algorithm (Yamany et al., 2015). It can improve the performance of the SVM training model and has the advantages of simplicity and efficiency. Ramakrishnan and Sankaragomathi (2017) used the modified region growing (MRG) and GWO to achieve the accurate segmentation of CT brain tumor images. Shankar et al. (2018) proposed an improved GWO to optimize the performance of multi-kernel SVM for thyroid disease classification.

In this paper, CEEMD was used to decompose the raw epileptic EEG signals into natural mode functions (IMF) of different frequencies, then these component signals were reconstructed and their linear and non-linear features were extracted. SVM-RFE was used to eliminate non-key features and reduce the influence of redundant features on recognition accuracy. Finally, the GWO-SVC classification model based on GWO optimized support vector classification (SVC) algorithm was applied to classify the EEG signals, which provided a theoretical basis for the aided diagnosis of epilepsy.

## Materials and Methods

### Selection of Experimental Data

The experimental data in this paper were from the EEG database of the epilepsy research center of the University of Bonn, Germany (Andrzejak et al., 2001). The sampling frequency of EEG signal acquisition system was 173.61 Hz, and the range of filtering bandwidth was 0.53–40 Hz. EEG data have been preprocessed to remove the artifacts and the data were widely used in public, so the experimental results have high reliability and contrast. The data set consists of five data subsets (denoted A–E), each of which contains 100 single-channel signals with a time of 23.6 s, and each single-channel signal contains 4,097 sampling points, and the bit of A/D conversion is 12 bits. The band-pass filter with a bandwidth of 0.53–40 Hz was used for filtering. Subsets A and B were EEG signals from the scalp surface of 5 healthy volunteers when they opened and closed their eyes, respectively. Subset C was the EEG signals of the hippocampal formations in five epileptic patients. Subset D was the EEG signal of the epileptogenic area with interictal epilepsy. Subset E was the EEG signal of the epileptogenic area during the ictal epilepsy.

The hardware condition of the computer used in the experiment was the Intel Core i7 processor, 4GB memory, win7 system. Under the environment of MATLAB r2016b (MathWorks, USA), the algorithm was used to simulate and test the data. The support vector machine chose the libsvm-mat-3.1 toolkit (Chang and Lin, 2011) to run.

### Complementary Ensemble Empirical Mode Decomposition

CEEMD is an improved signal decomposition method for EEMD proposed by Yeh et al. (2010). This method not only solves the problems of residual white noise and complex processing in EEMD (Wu and Huang, 2009) decomposition but also effectively suppresses the modal aliasing in the EMD decomposition method (Wu and Huang, 2010). The decomposition process of the CEEMD algorithm is based on EMD, adding a pair of auxiliary white noise with the same amplitude and opposite sign to the raw signals. These raw signals are decomposed into several intrinsic mode functions (IMFs) and residuals with clearer physical meaning. As the number of added noise increases, the residual amount of noise in reconstruction data will decrease, and the final residual amount can be almost ignored (Chen D. et al., 2018).

The decomposition steps of CEEMD are as follows:

*Step 1:* A pair of random Gaussian white noises with the same amplitude and opposite signs are added to the signal to form two new decomposition signals.

(1) $$\begin{array}{l} \{\begin{array}{c} S_{+i}\left(t\right)=S\left(t\right)+N_{i}^{+}\left(t\right)\\ S_{-i}\left(t\right)=S\left(t\right)+N_{i}^{-}\left(t\right) \end{array} \end{array}$$

Where *S*(*t*) is the raw signal, *N*_*i*_(*t*) is the white noise added for the *i* time, *S*_+*i*_(*t*) is the signal obtained by adding the positive white noise for the *i* time, and *S*_−*i*_(*t*) is the signal obtained by adding the negative white noise for the *i* time. Generally, the value is 0.01–0.5 times of the standard deviation of the original signal.

*Step 2:* EMD algorithm is used to decompose *S*_+*i*_(*t*) and *S*_−*i*_(*t*) to get their IMF components and residual terms.

(2) $$\begin{array}{l} \{\begin{array}{c} S_{+i}\left(t\right)=\overset{m}{\underset{j\;=\;1}{\sum }}I_{+ij}\left(t\right)+R_{+i}\left(t\right)\\ S_{-i}\left(t\right)=\overset{m}{\underset{j\;=\;1}{\sum }}I_{-ij}\left(t\right)+R_{-i}\left(t\right) \end{array} \end{array}$$

Where *I*_+*ij*_(*t*) denotes the *j* IMF component from *S*_+*i*_(*t*) decomposition, *I*_−*ij*_(*t*) denotes the *j* IMF component from *S*_−*i*_(*t*) decomposition, *R*_+*i*_(*t*) and *R*_−*i*_(*t*) denote the corresponding residual terms, respectively.

*Step 3: Step 1* and *step 2* are repeated for *m* times, and random white noise is added each time until the residual terms can no longer be decomposed.
*Step 4:* Calculate the mean value of IMF components obtained by decomposition, and take the mean value as the result of IMF component.

(3) $$\begin{array}{l} C_{j}\left(t\right)=\frac{1}{2m}\overset{m}{\underset{i\;=\;1}{\sum }}\left(I_{+ij}\left(t\right)+I_{-ij}\left(t\right)\right) \end{array}$$

where *C*_*j*_(*t*) denotes the first IMF component obtained by CEEMD.

### Support Vector Machine Recursive Feature Elimination

Support vector machine recursive feature elimination (SVM-RFE) is a feature selection method based on feature sorting technology proposed by Guyon et al. (2002). The function of RFE is to rank features by greedy strategy. Starting from the complete set, the least relevant features are eliminated one by one to complete the backward feature reduction, and finally, the optimal feature subset is obtained. SVM-RFE is a combination of SVM and RFE. In the process of SVM training, the weight of features can reflect their contribution to classification decision-making. Therefore, the weight of a classifier can be used as the basis of feature ranking, and then the relatively unimportant features are deleted one by one according to the weight of classifier until a certain number of features with higher importance are left. The combination of the SVM classification algorithm and feature selection process can improve the effectiveness of feature selection.

The steps of iterative reduction feature of SVM-RFE method are as follows:

*Step 1:* Input training sample data $D={\left\{d_{1},d_{2},...,d_{3}\right\}}^{T}$ and category label $L={\left\{l_{1},l_{2},...,l_{n}\right\}}^{T}$
*Step 2:* Initialize feature set α = {λ_1_, λ_2_, ..., λ_*n*_} and rearrange feature set β = {}
*Step 3:* The SVM classifier is used to train the input data, and the parameter information of the support vector is δ = *SVMtrain*(*D, L*)
*Step 4:* Calculate the cost function of features

(4) $$\begin{array}{l} f\left(x\right)=\frac{1}{2}D^{T}U\left(x\right)-\frac{1}{2}D^{T}U\left(-x\right) \end{array}$$

Where *U*(*x*) is a matrix with element *a*_*i*_*a*_*j*_*K*(*x*_*i*_, *x*_*j*_), *U*(−*x*) is the matrix after eliminating *x* features, and *K* denotes the kernel function of correlation between *x*_*i*_ and *x*_*j*_

*Step 5:* The weight coefficient *w* is used as the ranking criterion of feature importance to reorder new features. Get a new feature order set β = {β_1_, β_2_, ..., β_*n*_}, and remove the feature with the smallest weight coefficient from the current order set, repeat Step 3–Step 5, until enough features are deleted
*Step 6:* A set of nested feature subsets *Z*_1_⊂*Z*_2_⋯*Z*_*n*_ is defined, *Z*_*i*_(*i* = 1, 2, ⋯ , *n*) represents a subset of the top most important features selected from the feature set, and uses the recognition rate of the classifier as the evaluation index to select the best subset.

### Gray Wolf Optimizer Combine With Support Vector Classification

Gray wolf optimizer (GWO) is an advanced heuristic group intelligent optimization algorithm proposed by Mirjalili et al. (2014). This algorithm is mainly an optimized search method which simulates the social hierarchy of gray wolf and the way of preying on its prey. It has strong convergence performance, few parameters and easy to realize, and so on. SVM is originally a two classification model and can be used to solve multi-classification problems. It is a linear classifier with the largest interval defined in the feature space, which makes it different from the perceptron (Utkin et al., 2016). The learning strategy of SVM is to maximize the interval. SVM is a non-linear classifier in essence. SVM algorithm can be used for pattern classification or nonlinear regression, and SVC is the algorithm used by SVM to solve classification problems (Chen et al., 2010). The classification performance of the SVC model is affected by the penalty coefficient *c* and kernel function parameter *g*. Through the GWO algorithm, the SVC parameters are optimized to find the best classification parameters c and *g*, so as to obtain the GWO-SVC model with good performance.

The specific parameter optimization steps are as follows:

*Step 1:* α, β, and γ are three different classes of primitive wolves with the same scale generated from feasible region *W* = {*w*_1_, *w*_2_, ⋯ , *w*_*n*_}
*Step 2:* Initialize the position of the original wolves, obtain the fitness μ of gray wolf individuals in the population, and define the optimal and suboptimal fitness as *c* and *g*, respectively
*Step 3:* Select the fitness of the top three, and set the corresponding gray wolf to α, β, and γ in order
*Step 4:* Constantly move the position of gray wolf when it preys on prey and updates the subordinate wolves. The updating formula is as follows:

(5) $$\begin{array}{l} \{\begin{array}{c} Q_{\alpha }=|W\left(t\right)-H_{1}W_{\alpha }|\\ Q_{\beta }=|W\left(t\right)-H_{2}W_{\beta }|\\ Q_{\gamma }=|W\left(t\right)-H_{3}W_{\gamma }| \end{array} \end{array}$$

(6) $$\begin{array}{l} \{\begin{array}{c} W_{1}=W_{\alpha }-K_{1}Q_{\alpha }\\ W_{2}=W_{\beta }-K_{2}Q_{\beta }\\ W_{3}=W_{\gamma }-K_{3}Q_{\gamma } \end{array} \end{array}$$

(7) $$\begin{array}{l} \begin{array}{c} W\left(t+1\right)=\frac{1}{3}\left(W_{1}+W_{2}+W_{3}\right) \end{array} \end{array}$$

Where *W*_α_, *W*_β_, and *W*_γ_ denote the location of the gray wolf, and *H*_1_, *H*_2_, *H*_3_, *K*_1_, *K*_2_, and *K*_3_ are scale factors

*Step 5:* Update the values of α, *H*, and *K*. If the constraints are not met, go to *step 2*
*Step 6:* Use output parameters *c* and *g* to build SVC model for classification and recognition.

### Evaluation Index

The effect of a signal processing method is determined by the comparison of some digital evaluation indexes, such as pearson correlation coefficient (*Pr*), signal to noise ratio (*SNR*), and mean absolute error (*MAE*) (Ou-Yang et al., 2012). Generally, the larger the *Pr* value is, the greater the linear correlation between signals is. The larger the *SNR* value is, the more useful the restored signal is and the less the distortion is. The smaller the *MAE* value is, the better the effect of signal filtering is.

The expression of the *Pr*:

(8) $$\begin{array}{l} Pr=\frac{\overset{m}{\underset{i\;=\;1}{\sum }}\left(X_{i}-\bar{X}\right)\left(Y_{i}-\bar{Y}\right)}{\sqrt{\overset{m}{\underset{i\;=\;1}{\sum }}{\left(X_{i}-\bar{X}\right)}^{2}}\sqrt{\overset{m}{\underset{i\;=\;1}{\sum }}{\left(Y_{i}-\bar{Y}\right)}^{2}}} \end{array}$$

The expression of the *SNR*:

(9) $$\begin{array}{l} SNR=10\log_{10}\frac{\overset{m}{\underset{i\;=\;1}{\sum }}X_{i}^{2}}{\sqrt{\overset{m}{\underset{i\;=\;1}{\sum }}{\left(X_{i}-Y_{i}\right)}^{2}}} \end{array}$$

The expression of the *MAE*:

(10) $$\begin{array}{l} MAE=\frac{1}{m}\overset{m}{\underset{i\;=\;1}{\sum }}|X_{i}-Y_{i}| \end{array}$$

Where *X*_*i*_ is the original signal, *Y*_*i*_ is the processed signal, $\bar{X}$ is the average value of the signal, and $\bar{Y}$ is the standard deviation of the signal.

### Feature Extraction

Because the information contained in EEG is usually recessive, it is difficult to find all the rules through observation, so it is necessary to extract the features of EEG data. Because of its unique characteristics, EEG is different from other physiological signals, and the characteristics of different EEG are also different. The purpose of EEG feature extraction is to extract relatively effective feature indexes from many EEG features. At present, there are many EEG characteristics studied in the literature, such as mean, variance, standard deviation, range, fluctuation coefficient (Yuan et al., 2012), variation coefficient (Vinton et al., 2004), sample entropy (Arunkumar et al., 2018), kurtosis (Javidi et al., 2011) and skewness (Gandhi et al., 2012). In this paper, we extracted the above nine features from EEG signals for analysis.

## Results and Analysis

### Analysis of EEG Signal of Primary Epilepsy

**Figure 1:**
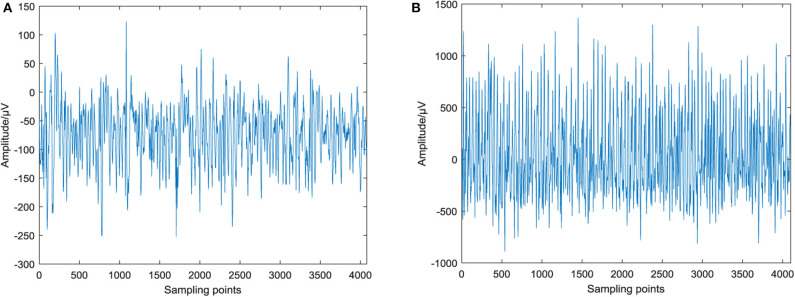
Raw EEG signal of epilepsy. **(A)** EEG signal of interictal epilepsy and **(B)** EEG signal of ictal epilepsy.

One single-channel signal is selected from subset D with interictal epilepsy and subset E with ictal epilepsy for waveform analysis. The raw epileptic EEG signal is shown in Figure 1, and the single-channel signal contains 4,097 sampling points. Figure 1A shows the EEG signal during the interictal epilepsy. The waveform of the signal is relatively stable with little fluctuation. The amplitude range is −252~123 μV. Figure 1B is the EEG signal during the ictal epilepsy, which fluctuates violently and has regularity. The amplitude range is −890~1,367 μV. The amplitude of EEG in the ictal period is obviously larger than that in the interval period, and the fluctuation gap is obvious, which indicates that the signal is excited and fluctuates violently in the ictal period. This phenomenon is consistent with the state of EEG activity with ictal epilepsy.

### CEEMD Based on Signal Evaluation Index

**Figure 2:**
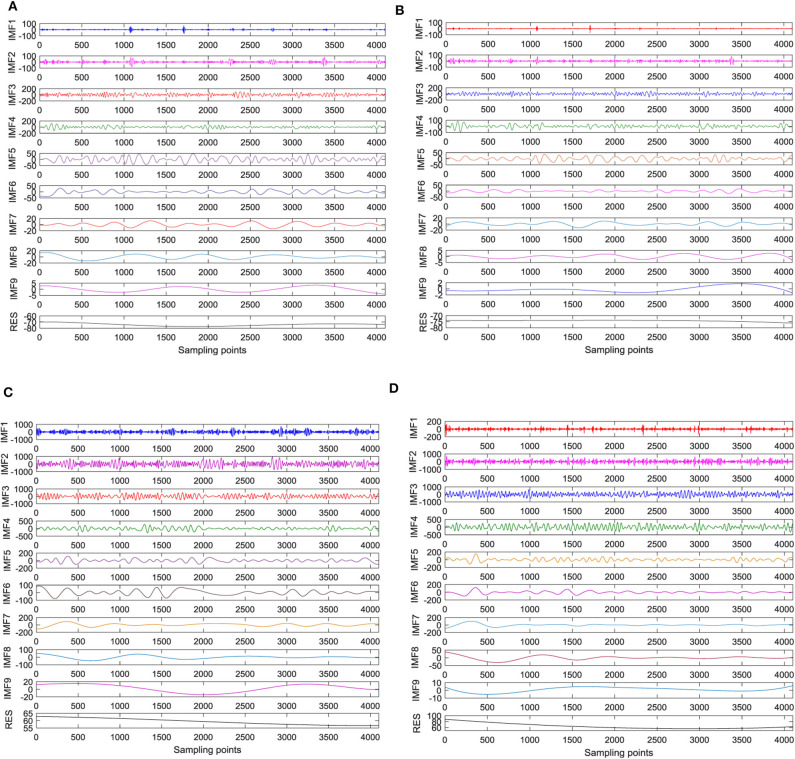
The signal decomposition of EEG between interictal and ictal period. **(A)** EMD decomposition during the interictal period, **(B)** CEEMD decomposition during the interictal period, **(C)** EMD decomposition during the ictal period, and **(D)** CEEMD decomposition during the ictal period.

EMD and CEEMD are used to decompose epileptic EEG signals, and the Intrinsic Mode Function (IMF) components of each order are obtained. Based on the MATLAB platform, the standard deviation of the added white noise is set to 0.2 times of the raw signal of the standard deviation. The number of iterations is set to 100, and the number of IMF is set to 9 (not including the trend). The signal decomposition of EEG between interictal and ictal period are shown in Figure 2. The raw EEG signal is decomposed into nine IMF and one residual term. The decomposed IMF components are arranged in the order of frequency from high to low, and each component has its own amplitude and frequency. With the increase of IMF component orders, the more stable the signal changes, the smaller the corresponding energy. The signal changes during the ictal period are more intense than during the interictal period. The amplitudes of the first four orders are larger than those of other orders. It can be seen from Figures 2A,C that the amplitude of IMF in each stage of ictal EEG signal processed by EMD is larger than that of the interictal EMD, and the difference is obvious. High-frequency signals with small amplitude appear in some sampling points of the first three IMF components, that is to say, there are different degrees of mode aliasing, which is more obvious in the ictal period. However, it can be seen from Figures 2B,D that there is no small-amplitude and high-frequency signals in the first three stages of EEG signals and seizure signals processed by CEEMD, which indicates that CEEMD can solve the problem of mode aliasing caused by EMD decomposition. There are great differences in amplitude and frequency between interictal EEG and ictal EEG.

**Figure 3:**
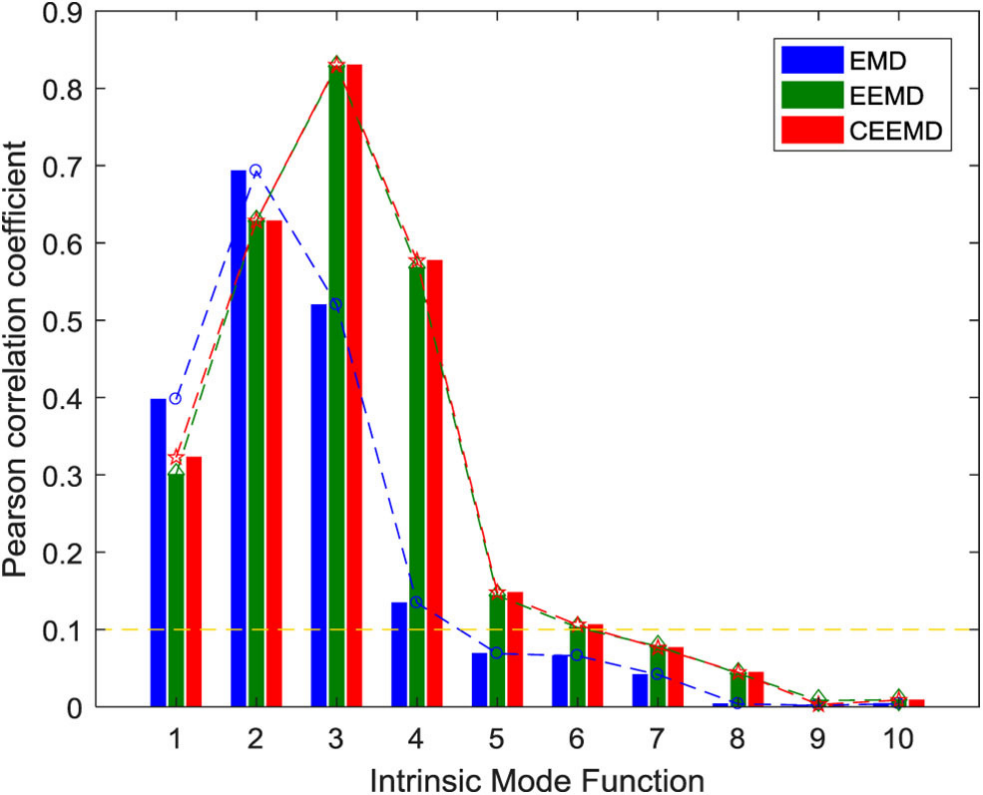
Correlation between IMF and raw signals in different stages of ictal period signals.

EMD, EEMD, and CEEMD are used to decompose the IMF component of the ictal period signals and conduct correlation analysis with the original signals, as shown in Figure 3. It can be concluded from the correlation property that the *Pr* of IMF2 and IMF3 decomposed by EMD is >0.5, which shows a strong correlation. The *Pr* of IMF2 reaches a maximum value of 0.6932, followed by a decreasing trend of IMF. The results of EEMD and CEEMD show that the *Pr* of IMF2, IMF3, and IMF4 are more than 0.5, which shows a strong correlation. The *Pr* of the two decomposition methods reach the maximum at IMF3, and their values are 0.8316 and 0.8300, respectively. The *Pr* of the latter IMF shows a decreasing trend. In addition to the first two IMF components, the *Pr* of the remaining eight IMF components decomposed by EMD are smaller than the *Pr* of the corresponding components decomposed by EEMD and CEEMD. The evaluation indexes of 10 IMF decomposed by different decomposition methods are shown in Table 1. The difference between the average *Pr* of the IMF decomposed by CEEMD and EEMD is very small and larger than that of EMD. The average *Pr* of EMD and EEMD is close, and both are smaller than the CEEMD decomposition method. CEEMD's average *MAE* is also smaller than the other two signal decomposition methods. In general, the CEEMD has relatively good signal evaluation indexes. However, from the signal evaluation index, it can be seen that the average *Pr* of different IMF decomposed by three methods is between 0.1 and 0.3, which shows weak correlation, indicating that a single IMF cannot represent all the information of the raw EEG signals. We need to select some useful IMF components for signal reconstruction in order to avoid the influence of distorted signals on the subsequent EEG recognition.

**Table 1:** Evaluation indexes of different decomposition methods.

**Method**	**Average Pr**	**Average SNR**	**Average MAE**
EMD	0.1964	0.4946	296.4282
EEMD	0.2701	0.4233	296.5334
CEEMD	**0.2745**	**0.7692**	**287.3643**

*Bold values indicate the best experimental results more intuitively*.

Generally, it is considered that *Pr* has no correlation in the range of 0–0.09. The threshold value is set to 0.1, IMF components below the threshold value are deleted, and the components above the threshold value are reserved for signal reconstruction. As can be seen from Figure 3, the IMF1–IMF4 components decomposed by EMD, the *Pr* of the IMF1–IMF6 components decomposed by EEMD and CEEMD are all higher than 0.1. We select these IMF components to reconstruct the EEG signals. The evaluation indexes reconstructed by different decomposition methods are shown in Table 2. After reconstruction, the evaluation indexes of EEG signals are better than those of a single IMF component signal. The *Pr* of reconstructed signals and raw signals are all >0.9, showing a strong correlation, which shows that signal reconstruction is a necessary job. In conclusion, CEEMD is better than the other two methods in decomposing and reconstructing the signals, and CEEMD is chosen as the preprocessing method of the raw EEG signals.

**Table 2:** Evaluation indexes reconstructed by different decomposition methods.

**Method**	**Pr**	**SNR**	**MAE**
EMD	0.9856	12.4403	72.8892
EEMD	0.9954	5.5925	158.0966
CEEMD	**0.9959**	**14.3365**	**67.1919**

*Bold values indicate the best experimental results more intuitively*.

**Figure 4:**
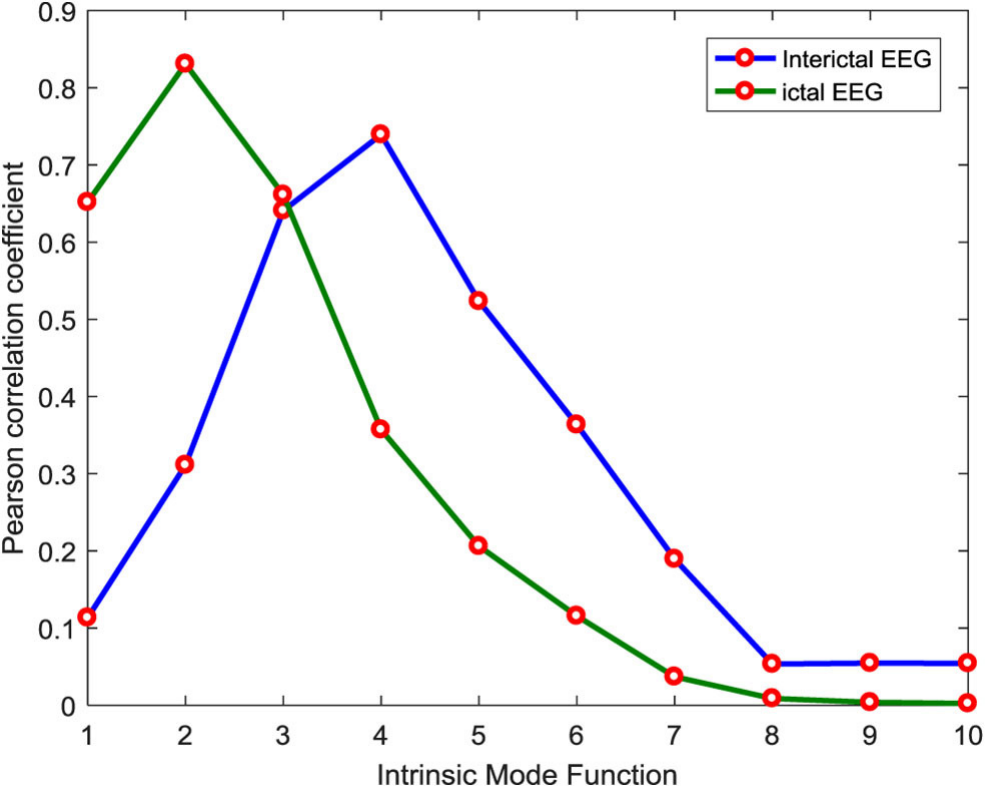
Correlation between IMF components of different channels and raw signals.

**Figure 5:**
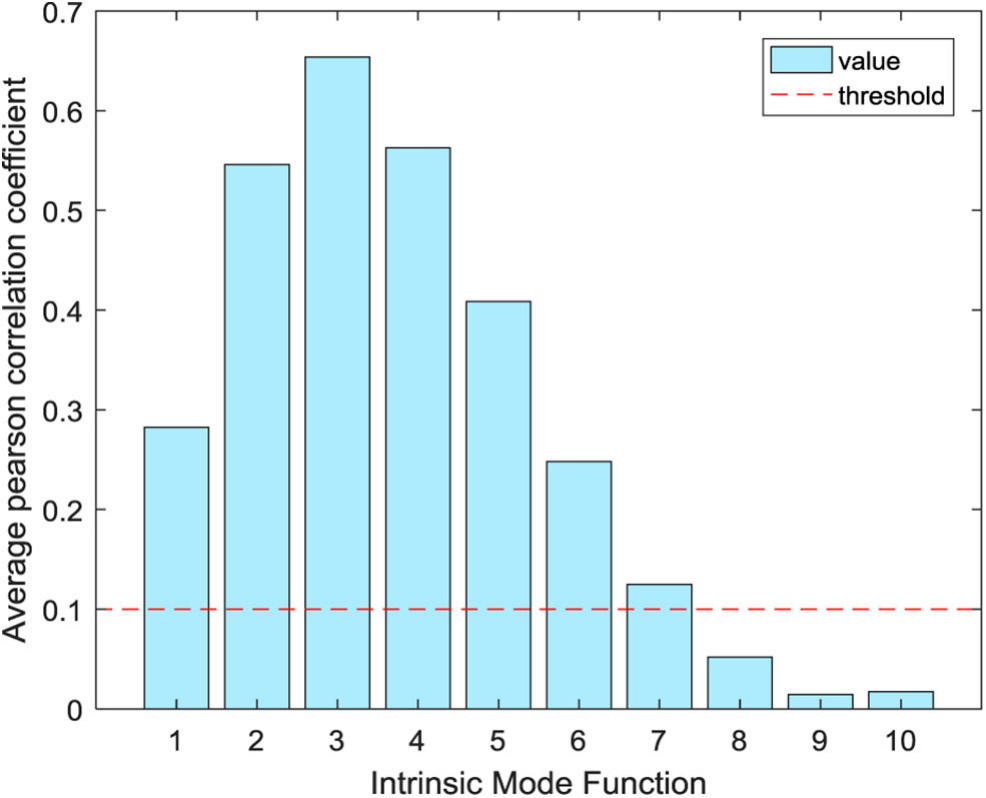
Correlation between IMF components of all channels and raw signals.

The above simulation experiment is to analyze the correlation of one channel of epileptic EEG during the ictal period, and the next is to analyze the correlation of two kinds of EEG signals during the interictal and ictal period. Each type of signal has 100 channels. Here, one channel is selected from the two types of signals for further correlation analysis. As shown in Figure 4, the maximum correlation component of EEG signals during the interictal period is IMF4, the maximum correlation IMF component of EEG signals during the ictal period is IMF2, and the maximum correlation IMF components of different types of signals are different. The EEG samples of 200 channels are decomposed by the CEEMD method, and the average *Pr* of IMF components is used as the division basis of useful signals. The threshold value is set to 0.1. As shown in Figure 5, the average *Pr* of IMF1–IMF7 components is higher than 0.1. Finally, we select these seven IMF components to reconstruct all EEG signals.

### Feature Extraction of EEG Signals

The feature extraction of 200 single-channel signals reconstructed from the interictal and ictal period is carried out. The extracted 9 features, namely mean, variance, standard deviation, range, fluctuation coefficient, variation coefficient, sample entropy, kurtosis, and skewness will be used in the next iterative feature reduction analysis. In the extraction of sample entropy, *m* = 2, *r* = 0.2 std. Because 9 features will produce many combinations of different feature subsets, it will lead to low training efficiency and model performance degradation. Therefore, the SVM-RFE algorithm should be used to rank the epileptic EEG data according to the weight of feature importance and select the combination of the optimal features.

### Reduction of Secondary Features and Establishment of Classification Models

**Figure 6:**
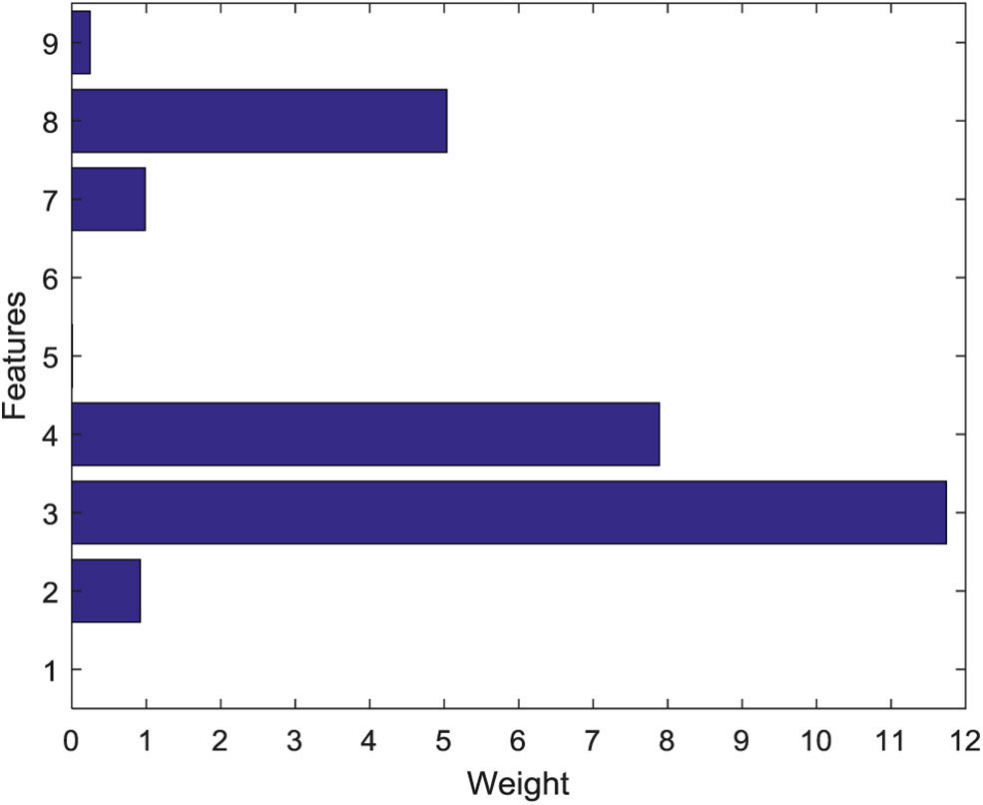
Weight values of different features.

When SVM-RFE is used to reduce the secondary features of data, it is necessary to normalize the data to [0,1] interval first to avoid the adverse effect of a too large difference between different features of data on the experimental results. Gaussian radial basis function (RBF) is used as a kernel function of SVM. The weight values of different features are shown in Figure 6. The sequence numbers 1–9 correspond to the nine features extracted from the EEG signals, respectively. This figure fully reflects that there are obvious differences in the importance of each feature of the EEG signals. It can be seen that the weight value of the standard deviation feature is the largest, indicating that the feature covers a lot of useful information on the EEG data. The weight of mean value, fluctuation coefficient, and variation coefficient is very small, which shows that the importance of these three characteristics is relatively low. According to the weight values of different features, the new features are sorted as {3,4,8,2,7,9,5,1,6}.

Because the first feature is the last one to be eliminated, it is also the most important feature. Therefore, based on all feature combinations in the new feature sorting, the features with the lowest importance in the current feature set are eliminated one by one feature at a time, and the number of features is reduced iteratively until it is reduced to the most important standard deviation feature. There are nine different feature sets. 80% (160) of 200 epileptic EEG signals are divided into training sets and the remaining 20% (40) into test sets. The data of different feature combinations in EEG signals are input to the GWO-SVC classification models in turn. The accuracy of the training set and test set obtained by a training classifier is used as the evaluation index of secondary feature reduction to select the optimal subset. In order to ensure the accuracy of classification results and the efficiency of recognition process at the same time, the initial number of the gray wolf is set to 20, the maximum number of iterations is set to 50, and the search interval of penalty coefficient and kernel function parameter is [0,100].

The classification accuracy of different feature subsets is shown in Table 3. The accuracy of the training set is on the decline, while the accuracy of the test set is on the rise and then on the decline. When the number of features in the feature subset is reduced from 9 to 8, the accuracy of the test set reaches the maximum of 100% for the first time, and only one channel EEG signal in the training set is misclassified. When the number of features is reduced to 6, the accuracy of the training set and the test set begins to decline. There are eight iterations until there is only one feature left. The purpose of secondary feature reduction is to improve the classification accuracy by filtering features or to reduce the dimension of feature set without reducing the classification accuracy. Although the accuracy of the training set of the full feature set is 100%, there are EEG signals in the test set which are misclassified, and the number of features is the most, which results in the low efficiency of model training. Finally, the subset {3,4,8,2,7,9} of six features with fewer features and higher classification accuracy is selected as the result of the SVM-RFE algorithm.

**Table 3:** Classification accuracy of different feature subsets.

**Feature subset**	**Feature numbers**	**Best c**	**Best g**	**Training set/%**	**Test set/%**
{3,4,8,2,7,9,5,1,6}	9	60.1100	6.9761	100 (160/160)	97.5 (39/40)
{3,4,8,2,7,9,5,1}	8	33.9487	8.6581	99.38 (159/160)	100 (40/40)
{3,4,8,2,7,9,5}	7	70.2355	5.2786	99.38 (159/160)	100 (40/40)
**{3,4,8,2,7,9}**	**6**	79.1905	7.7580	**99.38 (159/160)**	**100 (40/40)**
{3,4,8,2,7}	5	72.8569	9.2752	98.75 (158/160)	97.5 (39/40)
{3,4,8,2}	4	1.1068	70.1738	98.13 (157/160)	97.5 (39/40)
{3,4,8}	3	15.0708	12.2651	97.5 (156/160)	97.5 (39/40)
{3,4}	2	9.8490	88.9158	96.25 (154/160)	95 (38/40)
{3}	1	24.4377	39.4796	93.13 (149/160)	95 (38/40)

*Bold values indicate the best experimental results more intuitively*.

**Figure 7:**
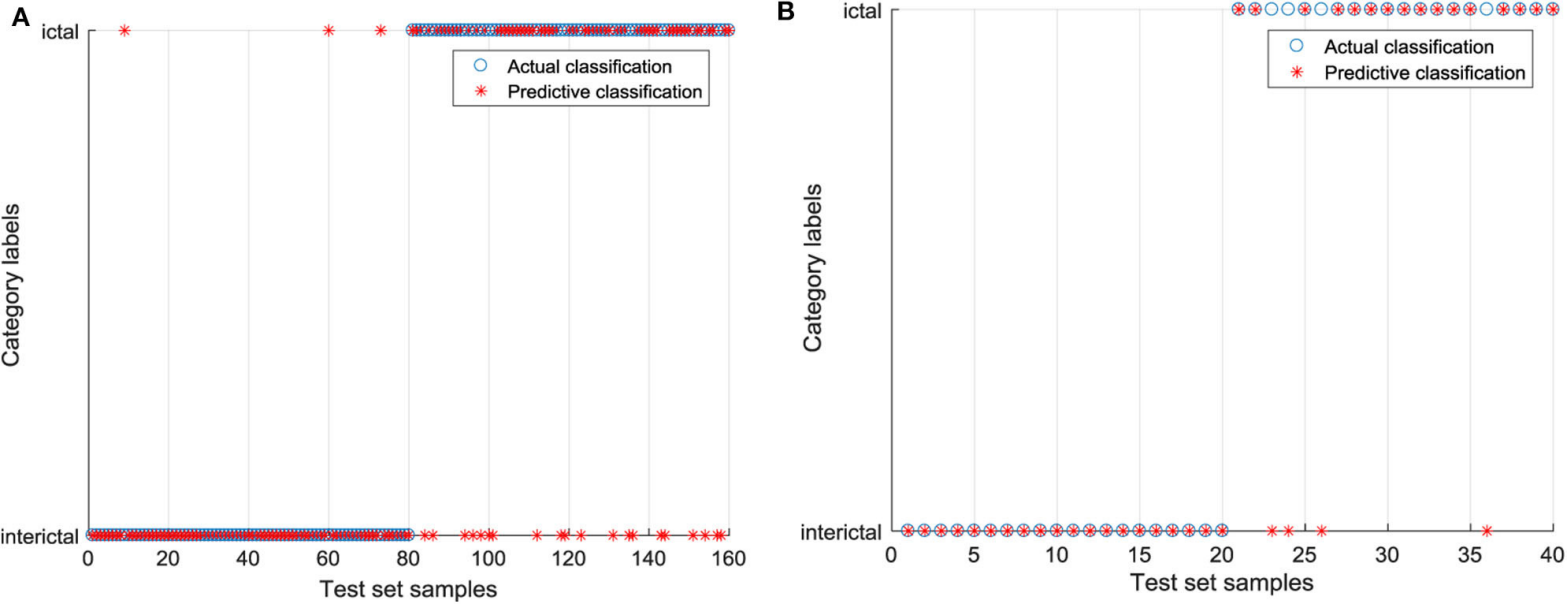
The classification results of the raw EEG signals by SVC. **(A)** Classification diagram of training set and **(B)** Classification diagram of test set.

**Figure 8:**
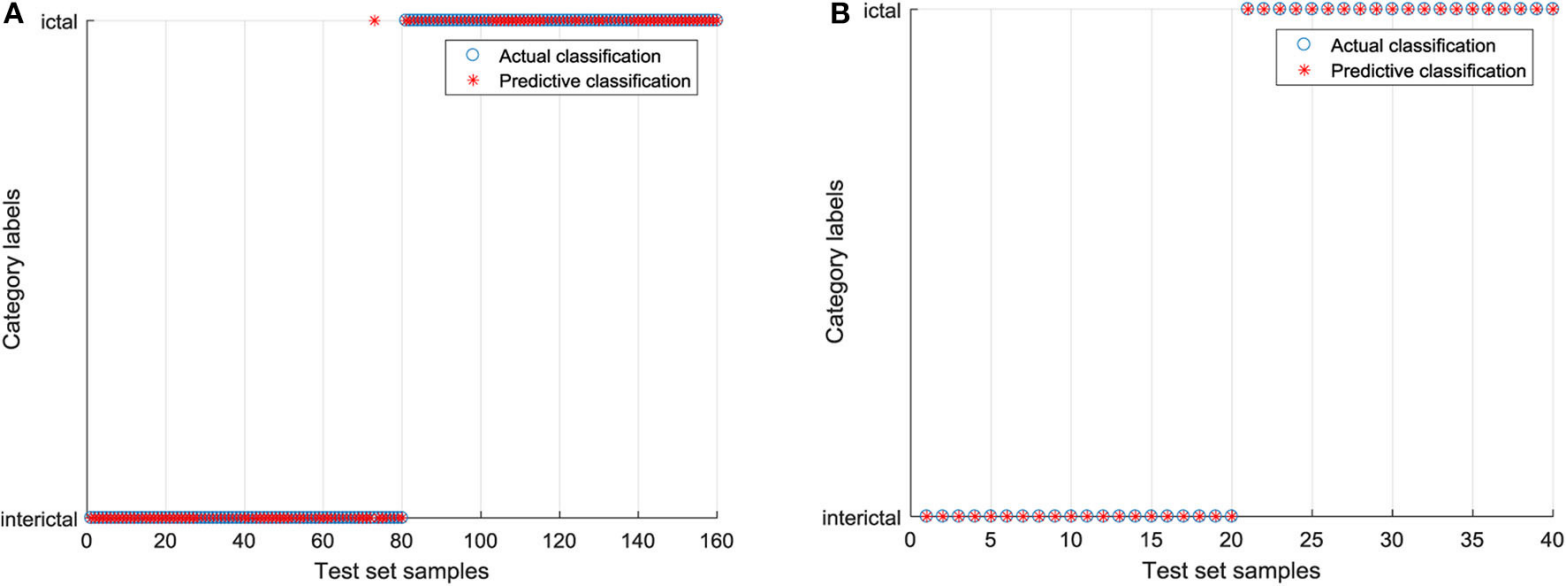
The EEG signals processed by CEEMD are classified by GWO-SVC. **(A)** Classification diagram of training set and **(B)** Classification diagram of test set.

Based on the 9 features extracted from the raw EEG data, the SVC model without parameter optimization is established, and RBF is chosen as the kernel function. In libsvm-mat-3.1 toolkit, the default value of a penalty coefficient *c* is 1, and the default value of a kernel function parameter *g* is the reciprocal of feature number (1/features). In order to clearly express the difference between the test category and the actual category, the blue “°” in the figure is the actual category of the input sample, and the red “*” is the predicted result of the classification model. If “°” and “*” coincide, the sample is correctly classified. The classification results of the raw EEG signals by SVC are shown in Figure 7. In the training set, 23 EEG signals were identified incorrectly, including three EEG signals in the interictal period and 20 EEG signals in the ictal period. A total of four EEG signals in the test set were identified incorrectly, and they were all EEG signals during the ictal period. The EEG signals processed by CEEMD are classified by GWO-SVC as shown in Figure 8. Only one EEG signal in the training set is identified incorrectly, which was the 73rd EEG signal in the interictal period. All the EEG signals in the test set are correctly identified. It can be seen that the training and test set of the GWO-SVC model established by the EEG signal after CEEMD processing has significantly better recognition results than the SVC classification model established by the unprocessed raw EEG signals. It shows that the method in this paper is applicable to the aided diagnosis of epileptic EEG, and it realizes the precise identification of EEG signals.

### Comparison With Other Methods

In order to verify the classification effect and superiority of the proposed method for epilepsy EEG recognition, the algorithm in this paper not only performs longitudinal comparative analysis and research with the SVC classification results of the unoptimized parameters of the raw EEG data but also compares with the classification results of grid search (GS), genetic algorithm (GA), particle swarm optimization (PSO), artificial bee colony (ABC), cuckoo search (CS), and firefly algorithm (FA) intelligent optimization algorithms. Other classifiers are similar to the GWO algorithm. The number of initial population is set to 20, the maximum number of iterations is set to 50, the search interval of penalty coefficient and kernel function parameters is [0,100], and the EEG data are normalized to [0,1] interval. Through such work, the unity of initial conditions can be ensured. Table 4 shows the classification results of different processing methods. It can be seen that the number of features selected by the model without parameter optimization and parameter optimization is different. The modeling time of SVC without parameter optimization is short, but the accuracy of the training set is low. It takes less time to establish the SVC model without parameter optimization, but the accuracy of the training set is low. CEEMD has little effect on the accuracy of the SVC model without parameter optimization. The accuracy of the training set and test set of GWO-SVC model is significantly higher than that of SVC. Compared with the raw EEG signals, the training set and test set accuracy of the model is improved after the signal is processed by CEEMD and SVM-RFE. Compared with the SVC model based on the raw EEG signals, the accuracy of training set classification and test set classification of the optimization model based on the algorithm in this paper is improved by 13.755 and 10%, respectively.

**Table 4:** Classification results of different treatment methods.

**Processing method**	**Feature numbers**	**Best c**	**Best g**	**Training set/%**	**Test set/%**	**Time (s)**
Raw+SVC	9	1	0.1111	85.625 (137/160)	90 (36/40)	0.0739
Raw+GWO-SVC	9	47.9615	34.1838	100 (160/160)	95 (38/40)	2.6507
Raw+SVM-RFE+SVC	5	1	0.2	86.875 (139/160)	95 (38/40)	0.0731
CEEMD+SVC	9	1	0.1111	85.625 (137/160)	90 (36/40)	0.0503
CEEMD+GWO-SVC	9	60.1100	6.9761	100 (160/160)	97.5 (39/40)	1.7907
CEEMD+SVM-RFE+ SVC	5	1	0.2	87.5 (140/160)	95 (38/40)	0.0443
CEEMD+SVM-RFE+GWO-SVC	**6**	79.1905	7.758	99.38 (159/160)	**100 (40/40)**	**1.6551**

*Bold values indicate the best experimental results more intuitively*.

The classification results of different optimization algorithms are shown in Table 5. The training set classification accuracy of the GS algorithm optimization model is the lowest, and the recognition time is long. Although the FA algorithm can make the classification accuracy of the training set reach 100%, the classification accuracy of the test set is less than GWO and ABC, and the recognition time is longer than GWO. The accuracy of the test set of GWO, ABC, and CS algorithm is 100%, and all EEG signals are recognized correctly. However, the recognition time of the GWO-SVC model is only 1.6551 s, which is obviously faster than that of ABC-SVC, and CS-SVC model, and 2.7512 s faster than PSO-SVC model which has the slowest recognition speed. Compared with other heuristic intelligent optimization algorithms, the GWO algorithm is more effective and reliable in parameter optimization of the SVC model, where *c* is 79.1905, *g* = 7.758.

**Table 5:** Classification results of different optimization algorithms.

**Modeling method**	**Feature numbers**	**Best c**	**Best g**	**Training set/%**	**Test set/%**	**Time (s)**
GS-SVC	6	5.6569	8	97.5 (156/160)	97.5 (39/40)	4.0977
GA-SVC	6	43.9056	3.1953	98.75 (158/160)	97.5 (39/40)	3.2152
PSO-SVC	6	5.3156	8.7895	98.13 (157/160)	97.5 (39/40)	4.4063
ABC-SVC	6	85.5963	6.0455	99.38 (159/160)	100 (40/40)	3.5328
CS-SVC	6	72.2167	8.4386	99.38 (159/160)	100 (40/40)	3.1746
FA-SVC	6	82.2227	20.4157	100 (160/160)	97.5 (39/40)	1.8642
**GWO-SVC**	6	79.1905	7.758	99.38 (159/160)	**100 (40/40)**	**1.6551**

*Bold values indicate the best experimental results more intuitively*.

## Discussion and Conclusions

In this study, we have proposed a new method based on CEEMD combined with iterative feature elimination for EEG of epilepsy aided diagnosis. The CEEMD signal decomposition algorithm was used to decompose the raw EEG signals into the IMF of different orders, and then feature extraction is carried out for the reconstructed signals. The SVM-RFE algorithm was used to reduce secondary features. Finally, the GWO-SVC classification and recognition model was established to realize the accurate and fast identification of Epileptic EEG. From the experimental analysis process and results, we can see that:

1. CEEMD algorithm based on correlation analysis can make the non-stationary EEG data stable, decompose the complex EEG signals into IMF components with practical physical significance, and solve the problems of mode aliasing. This algorithm is superior to the traditional EMD algorithm in various evaluation indexes.
2. SVM-RFE is used to filter the features of EEG signals, which can reduce the redundant information acquisition in the EEG data that has no internal relationship with the classification. The useful information of epileptic EEG signals is reflected by fewer features. The complexity of a training model is reduced, and the recognition efficiency and reliability of the classification model are improved.
3. The normalized data get rid of the influence of the big difference of sample data, speed up the optimal solution process, and improve the classification accuracy. The GWO-SVC epileptic EEG recognition model has a good classification accuracy. Combining CEEMD and SVM-RFE algorithm, it can make the classification accuracy higher than the recognition model of all features, and improve the performance and generalization ability of the model.
4. The algorithm in this paper can be applied to the aided diagnosis of epileptic EEG. This method can accurately and quickly identify the types of epileptic seizures. It has a certain theoretical guidance and promotion value for doctors to achieve the early diagnosis of epileptic diseases and take a reasonable epileptic treatment plan in the later stage.

The EEG data of epilepsy in the experiment were collected in the laboratory. The collection conditions are better than the actual clinical diagnosis conditions, and the interference is relatively small, but there may be many uncertain factors in the actual EEG analysis. In this study, 200 groups of sample data were tested and analyzed, but the actual clinical diagnosis needs to analyze a large number of data, which brings many challenges to the auxiliary diagnosis of Epilepsy EEG. The results show that although the method proposed in this paper has achieved high recognition accuracy, there are still wrong samples. How to overcome these difficulties will become the focus of the next research, and also the key to improving the recognition rate of epilepsy. We are going to add the disadvantageous factors in the experimental analysis to the future research work, expand the sample size of training data, and constantly improve and optimize the intelligent analysis algorithm to achieve perfect recognition accuracy.

## Data Availability Statement

Publicly available datasets were analyzed in this study. This data can be found here: http://epileptologie-bonn.de/cms/front_content.php?idcat=193&lang=3&changelang=3.

## Author Contributions

MZ conceived the study and supervised the study. MZ and KB developed the method and wrote the manuscript. KB and FH implemented the algorithms. KB and WL analyzed the data. All authors read and approved the final manuscript and content of the work.

## Conflict of Interest

The authors declare that the research was conducted in the absence of any commercial or financial relationships that could be construed as a potential conflict of interest.
